# Spheroid co-culture of BMSCs with osteocytes yields ring-shaped bone-like tissue that enhances alveolar bone regeneration

**DOI:** 10.1038/s41598-022-18675-x

**Published:** 2022-08-27

**Authors:** Ying-Hui Zhou, Yue Guo, Jia-Yu Zhu, Chen-Yi Tang, Ya-Qiong Zhao, Hou-De Zhou

**Affiliations:** 1 National Clinical Research Center for Metabolic Diseases, Hunan Provincial Key Laboratory of Metabolic Bone Diseases, and Department of Metabolism and Endocrinology, The Second Xiangya Hospital of Central South University, Changsha, 410011 Hunan China; 2 Department of Stomatology, The Second Xiangya Hospital, Central South University, Changsha, 410011 Hunan China; 3 Department of Nutrition, The Third Xiangya Hospital of Central South University, Changsha, 410013 Hunan China

**Keywords:** Biomaterials - cells, Implants, Regeneration

## Abstract

Oral and maxillofacial bone defects severely impair appearance and function, and bioactive materials are urgently needed for bone regeneration. Here, we spheroid co-cultured green fluorescent protein (GFP)-labeled bone marrow stromal cells (BMSCs) and osteocyte-like MLO-Y4 cells in different ratios (3:1, 2:1, 1:1, 1:2, 1:3) or as monoculture. Bone-like tissue was formed in the 3:1, 2:1, and 1:1 co-cultures and MLO-Y4 monoculture. We found a continuous dense calcium phosphate structure and spherical calcium phosphate similar to mouse femur with the 3:1, 2:1, and 1:1 co-cultures, along with GFP-positive osteocyte-like cells encircled by an osteoid-like matrix similar to cortical bone. Flake-like calcium phosphate, which is more mature than spherical calcium phosphate, was found with the 3:1 and 2:1 co-cultures. Phosphorus and calcium signals were highest with 3:1 co-culture, and this bone-like tissue was ring-shaped. In a murine tooth extraction model, implantation of the ring-shaped bone-like tissue yielded more bone mass, osteoid and mineralized bone, and collagen versus no implantation. This tissue fabricated by spheroid co-culturing BMSCs with osteocytes yields an internal structure and mineral composition similar to mouse femur and could promote bone formation and maturation, accelerating regeneration. These findings open the way to new strategies in bone tissue engineering.

## Introduction

Bone defects in the oral and maxillofacial region caused by dental treatments (such as tooth extraction) and surgical resection of neoplasms may severely impair cosmetic appearance and oral function^1^. A critical-sized bone defect will not heal spontaneously and requires surgical reconstruction^2^, so bone substitutes remain an urgent need. The main sources of bone transplantation for clinical use include autologous bone transplantation, allogenic or heterologous bone transplantation, and tissue-engineered construction^3^. Autogenous bone grafts are widely regarded as the “gold standard,” but recipient tissue availability and donor site morbidity limit this approach^4–7^. Allografts and xenografts have clear limitations, including immunological rejection, premature resorption, infection risk, and lack of osteoinductive and angiogenic potential^8,9^. Therefore, tissue engineering has emerged as an attractive and alternative approach for bone regeneration^10^. This method targets the generation of biological and living substitutes for damaged tissue to restore, maintain, or improve tissue function^11,12^, but an ideal material for bone tissue engineering is lacking.

Biomaterials for bone regeneration have been developed from inert materials that cannot interact with physiological tissue, but current iterations include bioactive materials that can stimulate osteoblasts^13^. The biomaterials ideally should be able to promote osteogenic differentiation in vitro and bone formation in vivo^13,14^. Three-dimensional (3D) cell culture systems are increasingly used in the field because of its obvious advantages in providing more complex information about the physiology of the tissue^15^. One promising 3D method is the spheroid culture system, which facilitates cell–extracellular matrix (ECM) and cell–cell interaction and provides a physiochemical environment similar to the in vivo experience^16^. Spheroid-cultured cells are reported to have improved cell survival, stemness, multi-differentiation potential, and intrinsic phenotypic properties in vitro, as well as enhancing anti-inflammatory and angiogenic responses and bone formation in vivo^16,17^. This culture method has been widely used in cancer research, drug screening, embryonic development studies, clinical studies, and tissue engineering^16,17^. As an example, Moritani et al. compared nodule formation and expression of osteogenesis-related genes between monolayer-cultured human periodontal ligament (hPDL) mesenchymal stem cells (MSCs) and spheroid-cultured hPDLMSCs. They found significant enhancement of both outcomes with the spheroid-cultured hPDLMSCs and showed that transplantation of these spheroids could significantly promote new bone formation in a mouse model of a calvarial defect^18^. In an unrelated study using a rat model of critical-sized femoral segmental defects, spheroid-cultured MSCs showed increased survival, osteogenic potential, and vascular endothelial growth factor secretion, in vitro findings that were associated with improved bone healing with implantation^19^. These indicate that spheroid-cultured cells are promising and useful bioactive materials for bone regeneration.

The selection of seed cells is important in bone regeneration. MSCs in bone marrow, umbilical cord blood, and adipose tissue have a considerable ability to regenerate bone tissue^20^. Among these options, bone marrow stromal cells (BMSCs) have been suggested as an ideal seed cell source^21^. They also are the most frequently investigated type of MSCs for bone regeneration because they have the potential for multi-directional differentiation and are easy to obtain and expand^21^. BMSCs can differentiate into the osteogenic lineage and form bone-like tissues both in vivo and in vitro^22–24^ and have been used in the clinical treatment of osteonecrosis, total joint arthroplasty, and cartilage defect repair^21^. In addition, Kaigler et al. found that implantation of BMSCs with a gelatin sponge could accelerate bone regeneration in teeth extraction sockets when compared with a saline-soaked gelatin sponge^25^. In a phase I/II clinical trial, the use of BMSCs with a biodegradable 3D-poly-lactic-acid–based scaffold in periodontitis patients with intrabony defects resulted in clinically and radiographically significant defect improvement compared with the use of conventional periodontal surgical procedures without application of BMSCs^26^.

An excellent outcome for regenerative medicine also depends on cell survival and further differentiation^27,28^. However, BMSCs differentiation into osteoblasts and subsequently into DMP-1 expressing osteocyte-like cells is not spontaneous and requires an appropriate microenvironment^29^. Many cell types in bone tissue, including osteocytes, osteoblasts, and fibroblasts, can secrete ECM to form a natural and precisely arranged fibrous network that provides a specialized local microenvironment for tissue engineering^27^. Among these options, osteocytes are the most abundant cell type in bone and the master orchestrators of bone physiology and homeostasis^7,30,31^. We previously showed that osteocytes of 12-month *Irs-1*-null mice express higher alkaline phosphatase (ALP) than osteocytes of wild-type mice, enhancing bone formation and improving bone mineral density^29^. Osteocytes not only can regulate osteogenic differentiation through gap junctions^32^ but also can promote MSC recruitment, proliferation, and osteogenic differentiation by secreting factors^31^. Meanwhile, osteocytes are more influential than osteoblasts in stimulating osteogenesis in BMSCs. On their own, osteocytes can direct BMSCs into an osteoblast lineage, without the need for their extracts in a co-culture system, as can osteocyte-conditioned medium^33^. Thus, the co-culture of BMSCs with osteocytes may promote osteogenic differentiation for bone regeneration.

The aim of this study was to develop bioactive materials for alveolar bone regeneration. We used the spheroid culture method to co-culture BMSCs with osteocytes to fabricate bone-like tissue. The internal structure and mineral composition of the bone-like tissue were analyzed and compared with that of the mouse femur. Moreover, bone-like tissue was implanted into a mouse tooth extraction model to assess the effect of this bone-like tissue on bone regeneration in vivo.

## Results

### Stable ring-shaped bone-like tissues formed in the 3:1 co-culture group

**Figure 1:**
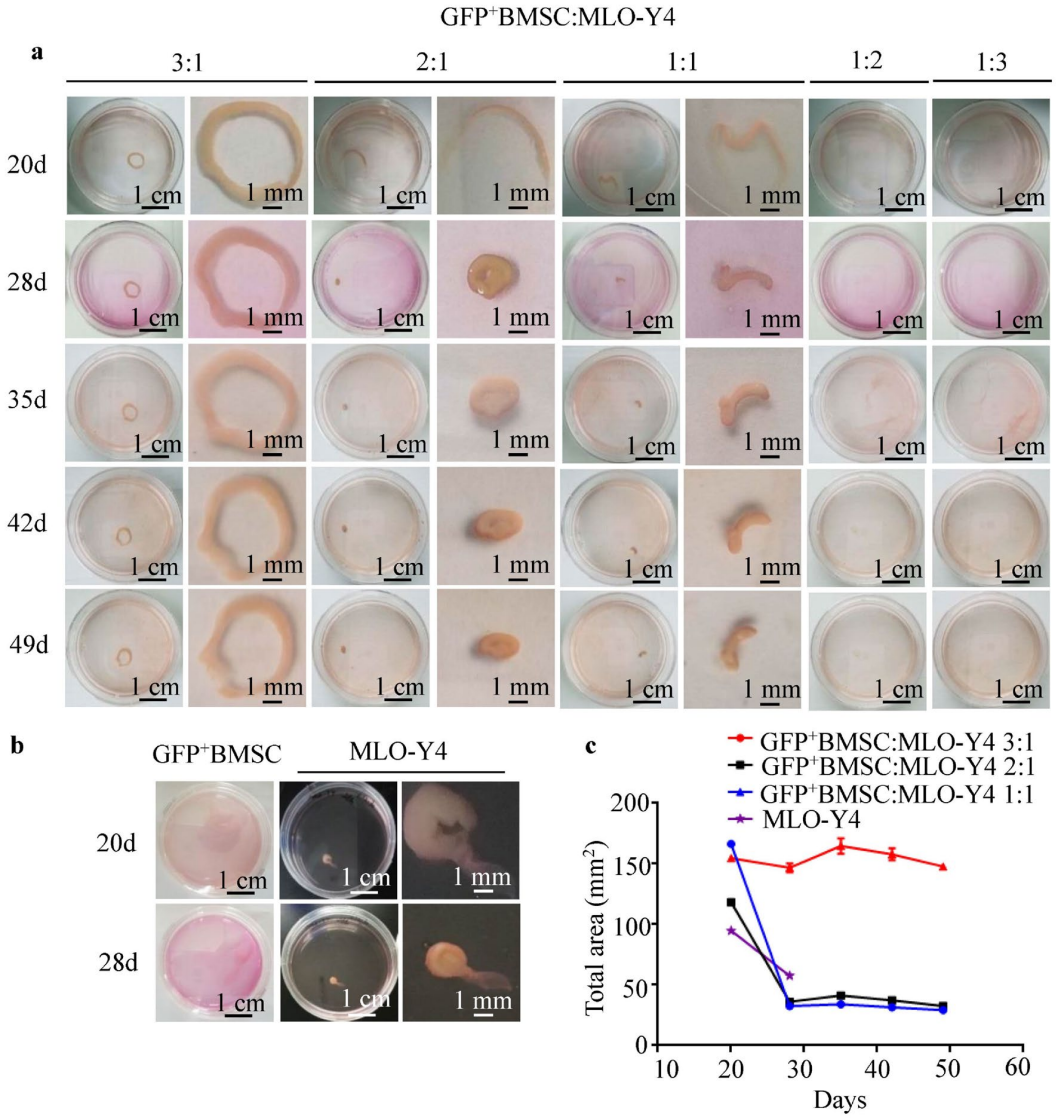
The formation of bone-like tissues. (**a**) GFP^+^BMSCs were co-cultured with MLO-Y4 cells in different proportions (3:1, 2:1, 1:1, 1:2, 1:3) for 20, 28, 35, 42, and 49 days. The 3:1, 2:1, and 1:1 ratios yielded bone-like tissues, whereas the 1:2 and 1:3 ratios did not. (**b**) The MLO-Y4 monoculture formed bone-like tissue, but the GFP^+^BMSCs did not. (**c**) The area of bone-like tissue decreased significantly in the MLO-Y4 monoculture and with the 2:1 and 1:1 ratios on day 28, but the area of bone-like tissue with the 3:1 ratio showed no significant change with extension of culture time.

Green fluorescent protein (GFP)-labeled BMSCs (GFP^+^BMSCs) and MLO-Y4 cells were spheroid cultured alone or co-cultured in different ratios (3:1, 2:1, 1:1, 1:2, 1:3) for 20, 28, 35, 42, and 49 days to allow the formation of bone-like tissues (Fig. 1). The 3:1, 2:1, and 1:1 co-cultures and MLO-Y4 monoculture formed bone-like tissues on day 20, whereas the 1:2 and 1:3 co-cultures and GFP^+^BMSC monoculture did not (Fig. 1a,b). The only ring-shaped bone-like tissues were formed naturally in the 3:1 co-cultures, which also yielded the largest area of bone-like tissues (Fig. 1c). The bone-like tissues formed at the 2:1 and 1:1 ratios and with the MLO-Y4 monoculture were significantly reduced in size by day 28 and detached from the bottom of the culture dishes on days 49, 49, and 28, respectively. However, the bone-like tissues formed in the 3:1 co-culture showed no significant change in size with prolonged culture and remained firmly attached to the bottom of the dish on day 49 (Fig. 1c).

### Bone-like tissues formed with the 3:1 co-culture of GFP^+^BMSCs:MLO-Y4 cells are the most similar to mouse femur

**Figure 2:**
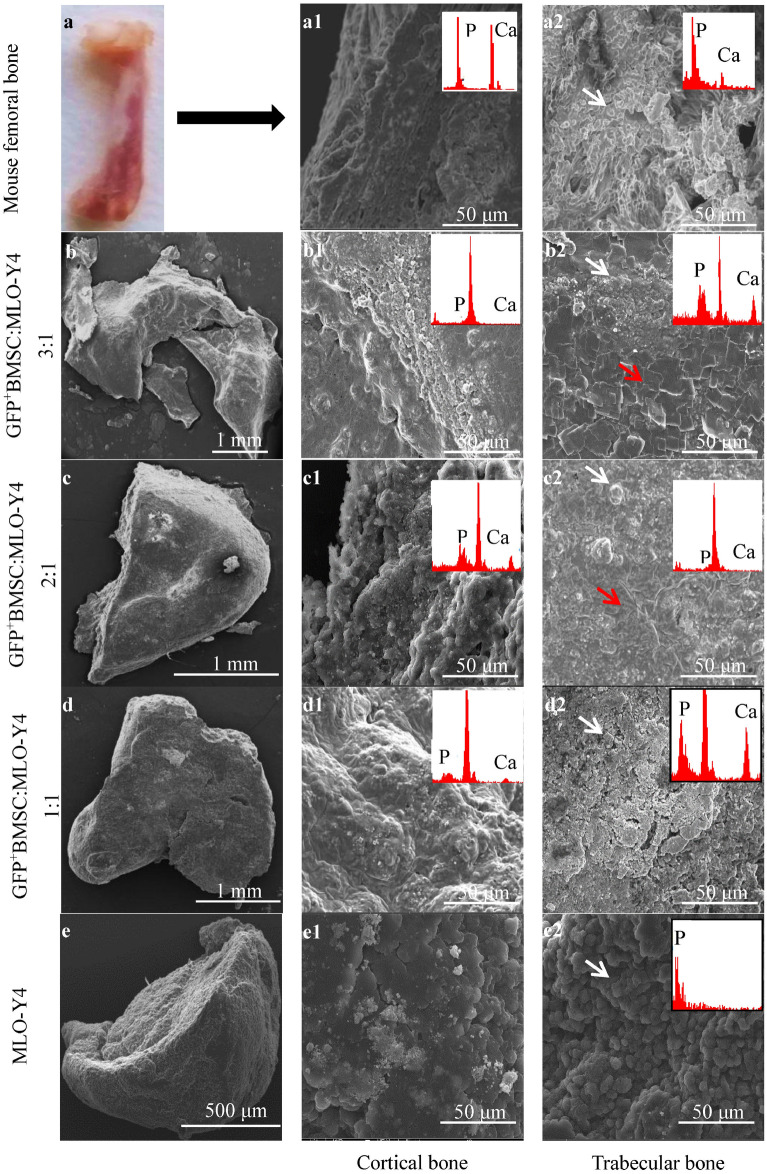
SEM morphology of the cross-sections of bone-like tissues and mouse femoral bone. Continuous dense layers of deposited calcium phosphate and ECMs were observed in the mouse femoral cortical bone (**a1**) and with GFP^+^BMSC:MLO-Y4 3:1 (**b1**), 2:1 (**c1**), and 1:1 (**d1**), but not with the MLO-Y4 monoculture (**e1**). Spherical calcium phosphate was observed in mouse femoral trabecular l bone (**a2**) and all of the spheroid cultures (**b2**–**e2**, white arrowheads). Flake-like calcium phosphate was present in the 3:1 (**b2**) and 2:1 (**c2**) co-cultures (red arrowheads). SEM, scanning electron microscopy.

We used scanning electron microscopy (SEM) to observe and compare the morphology of the bone-like tissues formed in spheroid culture with the cortical and trabecular bone of mouse femur (Fig. 2). At the cross-section of mouse femur, continuous dense layers of deposited calcium phosphate and ECM were observed in cortical bone (Fig. 2a1), and aggregates of spherical calcium phosphate were observed in trabecular bone (Fig. 2a2). Similar continuous layer structures were observed in the bone-like tissues of the 3:1, 2:1, and 1:1 co-cultures, but not in the bone-like tissues of the MLO-Y4 monoculture; however, the layer structures observed in the bone-like tissues were less dense than mouse femoral cortical bone (Fig. 2b1,c1,d1,e1). Meanwhile, similarly spherical calcium phosphate was observed in the cross-section of bone-like tissues of all groups (Fig. 2b2,c2,d2,e2, white arrowheads). Moreover, flake-like calcium phosphate was present in the bone-like tissues developing at the 3:1 and 2:1 ratios (red arrowheads).

The elemental composition of the bone-like tissues and mouse femoral bone was detected by SEM with energy dispersive X-ray spectroscopy (EDX, Table 1 and Fig. 2). Both phosphorus (P) and calcium (Ca) signals were observed in the bone-like tissues from the 3:1, 2:1, and 1:1 co-cultures. The highest concentrations of P and Ca were obtained in the trabecular-like structure of the 3:1 co-culture. Additionally, the Ca concentration in the trabecular-like structure of the 3:1 co-culture was lower than in femoral cortical bone but higher than in femoral trabecular bone. However, only the P signal was observed in the bone-like tissue from the MLO-Y4 monoculture, and no Ca signal was detected (Table 1).

**Table 1:** The elemental concentrations of bone-like tissues and mouse femoral bone expressed in mass%.

Element	BMSC:MLO-Y4 = 3:1		BMSC:MLO-Y4 = 2:1		BMSC:MLO-Y4 = 1:1		MLO-Y4		Femoral bone	
	a1	a2	b1	b2	c1	c2	d1	d2	Cortical bone	Trabecular bone
C	51.12	71.02	59.15	44.48	59.79	56.84	38.84	62.84	61.90	67.22
O	27.22	8.95	14.62	9.31	14.77	14.41	12.46	17.50	14.05	22.71
P	2.74	3.85	1.92	2.31	2.48	3.73	0.00	4.70	3.79	4.66
Ca	3.45	5.92	2.51	2.93	3.34	4.40	0.00	0.00	15.78	2.05
Others	15.47	10.26	21.80	40.97	19.62	20.62	48.30	14.96	4.48	3.36
Total	100.00	100.00	100.00	100.0	100.00	100.00	100.00	100.00	100.00	100.00

*C* carbon, *O* oxygen, *P* phosphorus, *Ca* calcium, *a1, b1, c1, and d1* cortical-like structure, *a2, b2, c2, and d2* trabecular-like structure.

### GFP^+^BMSC-derived osteocyte-like cells are positive for DMP1 and negative for ALP and Col1 in bone lacunae of bone-like tissues

**Figure 3:**
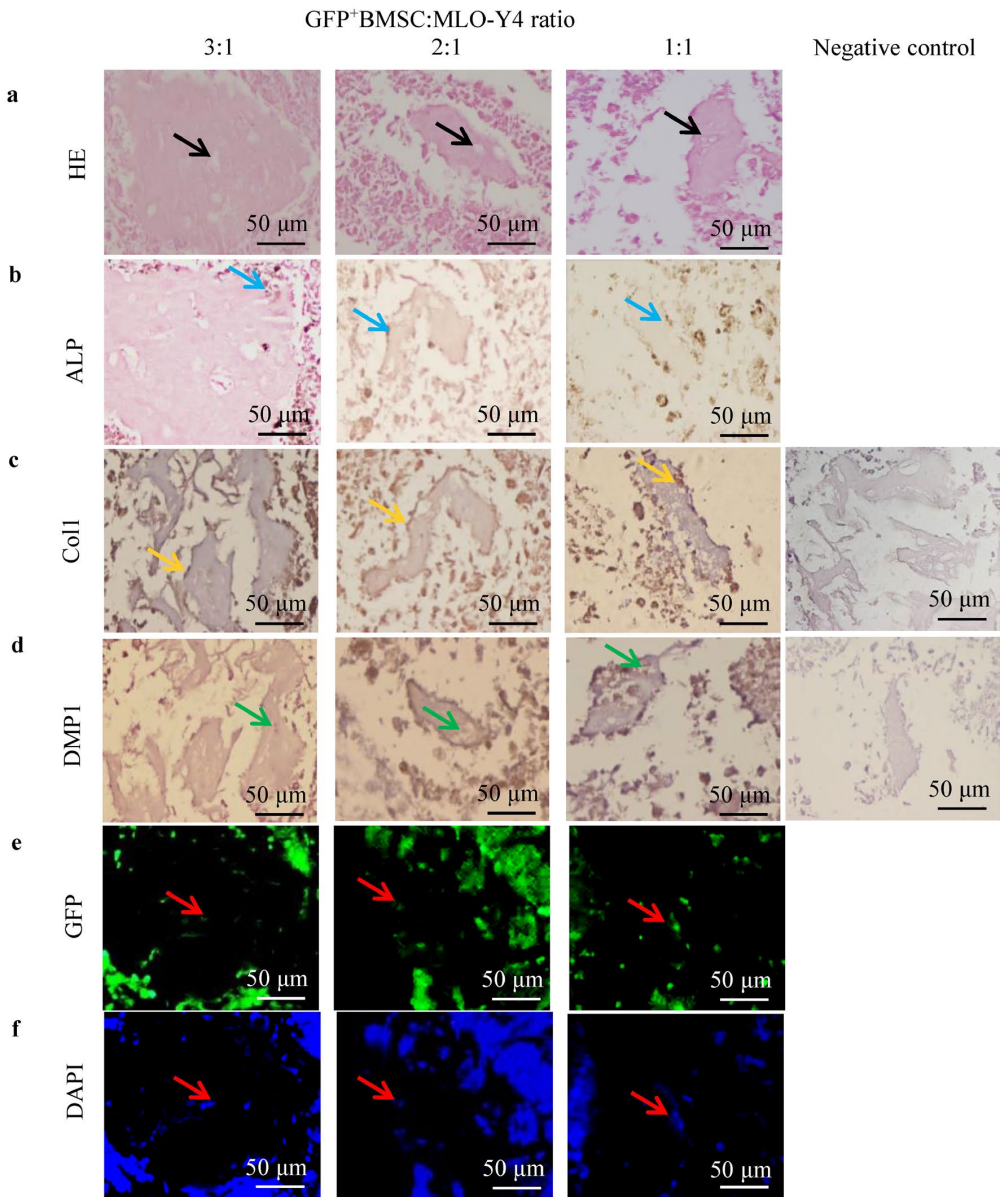
The histomorphological and immunohistochemical features of the bone-like tissues. (**a**) HE staining showed osteoblast-like cells surrounded by a cohesive osteoid-like matrix and osteocyte-like cells embedded into lacunae-like structures (black arrowheads) in the 3:1, 2:1, and 1:1 co-cultures. (**b**,**c**) Osteoblast-like cells on the osteoid-like matrix surface were positive for ALP (blue arrowheads) and Col1 (orange arrowheads). (**d**) Osteocyte-like cells in the osteoid-like matrix were positive for DMP1 (green arrowheads). (**e**,**f**) Fluorescence microscopy showed that osteocyte-like cells embedded in the osteoid-like matrix and osteoblast-like cells on the surface were GFP positive (red arrowheads). DAPI, 4′,6-diamidino-2-phenylindole.

We further evaluated the degree of osteogenic differentiation of bone-like tissues by HE and ALP staining and by type I collagen (Col1) and dentin matrix protein 1 (DMP1) immunohistochemistry. GFP positivity was used to confirm the cell source of the bone-like tissues (Fig. 3). HE staining of the bone-like tissues showed areas of lacunae-like structures (black arrowheads) surrounded by a cohesive osteoid-like matrix in the 3:1, 2:1, and 1:1 co-cultures (Fig. 3a), but not in the MLO-Y4 monoculture (data not shown). The size of the osteoid matrix in the 3:1 co-culture was larger than with the 2:1 or 1:1 ratio. The cells embedded in the lacunae-like structures of the cohesive osteoid-like matrix exhibited an osteocyte-like morphology. In cross-sections of bone-like tissues from the 3:1, 2:1, and 1:1 co-cultures, osteocyte-like cells in the osteoid-like matrix were positive for DMP1 (Fig. 3d, green arrowheads) and negative for ALP and Col1. Osteoblast-like cells on the osteoid-like matrix surface were positive for ALP (Fig. 3b, blue arrowheads) and Col1 (Fig. 3c, orange arrowheads). The osteocyte-like cells embedded in the osteoid-like matrix (black arrowheads) and the osteoblast-like cells on the osteoid-like matrix surface were GFP positive (Fig. 3e,f), and unembedded cells around the osteoid-like matrix surface were partially GFP positive.

### Bone-like tissue of the GFP^+^BMSC:MLO-Y4 3:1 co-culture accelerates bone regeneration of tooth extraction sockets

**Figure 4:**
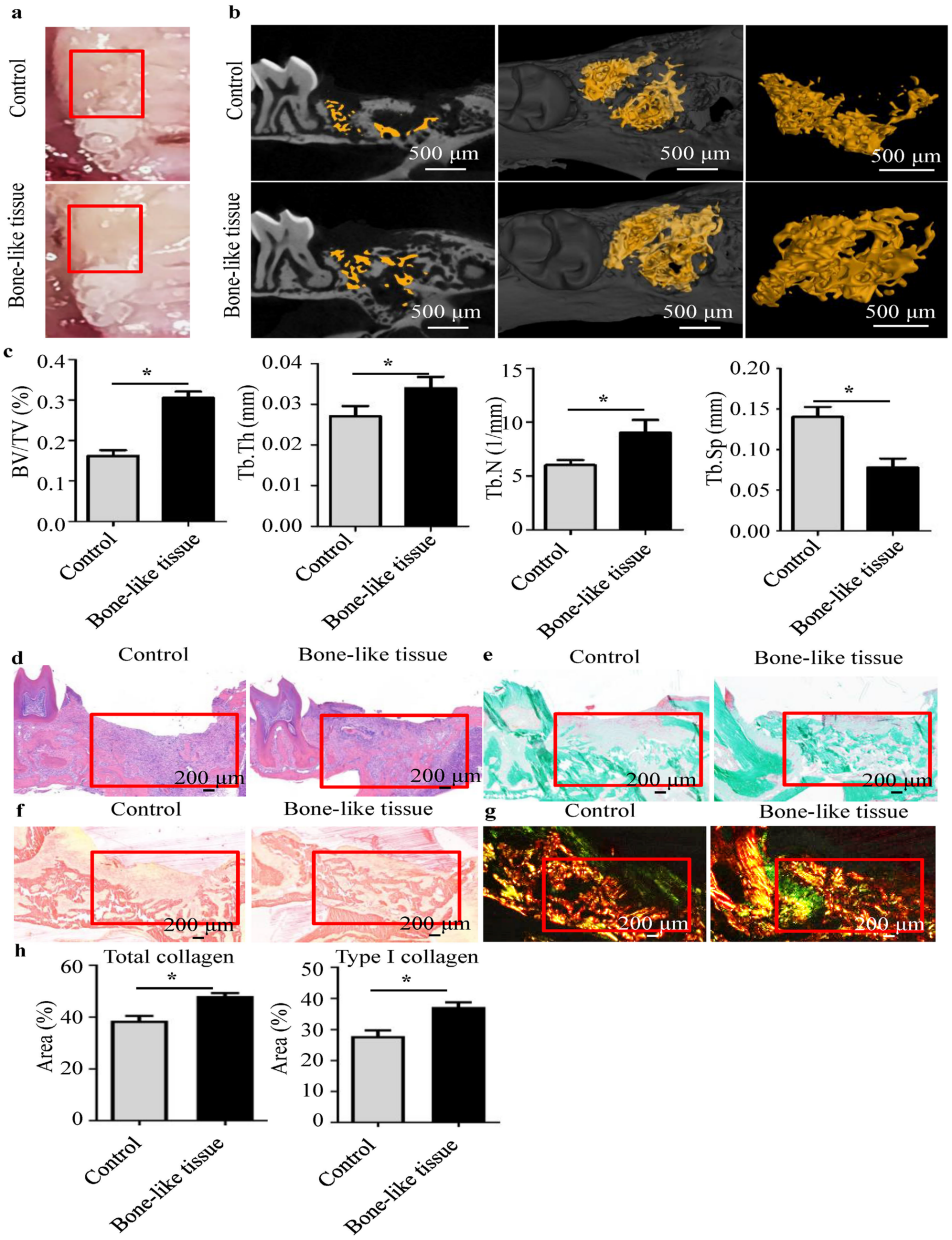
Transplantation of bone-like tissue in a tooth-extraction mouse model. (**a**) The extraction sockets in the bone-like tissue implantation group showed better healing (red box area). Micro-CT (**b**,**c**) and HE staining (red box area) (**c**) of the extraction sockets showed that the bone mass in the bone-like tissue implantation group was higher than in unimplanted controls. BV/TV, bone volume/total volume; Tb.Th, trabecular thickness; Tb.N, trabecular number; Tb.Sp, trabecular separation. (**d**) The extraction sockets of the bone-like tissue implantation group had more osteoid and mineralized bone by Goldner’s trichrome staining (red box area). Ordinary white-light microscopy (**e**) and polarized light microscopy (**f**) showed picrosirius red staining for the collagen fibers in the extraction sockets. (**g**,**h**) The bone-like tissue implantation group had more total collagen and type I collagen (red) than unimplanted controls. ^*^P < 0.05.

We transplanted bone-like tissues from the 3:1 co-culture into a tooth-extraction mouse model and evaluated bone regeneration of sockets at 2 weeks (Fig. 4). Healing in the bone-like tissue implantation group was better than in the unimplanted control group (Fig. 4a). Microcomputed tomography (micro-CT) imaging showed that the bone-like tissue implantation group had enhanced bone regeneration compared with the unimplanted group (Fig. 4b). The fraction of bone volume/total volume (BV/TV), trabecular thickness (Tb.Th) and trabecular number (Tb.N) also were higher with implantation, whereas trabecular separation (Tb.Sp) was lower (Fig. 4c). We found the same trend in the histomorphological analysis of the extraction sockets. Hematoxylin–eosin (HE) staining revealed a higher bone mass in the bone-like tissue implantation group (Fig. 4d). Meanwhile, Goldner’s trichrome staining showed more osteoid and mineralized bone in the extraction sockets of the bone-like tissue implantation group (Fig. 4e). Picrosirius red staining revealed that the bone-like tissue implantation group had more collagen than the unimplanted controls (Fig. 4f). Under polarized light microscopy, type I collagen was red and type III collagen was green (Fig. 4g)^34^. In the bone-like tissue implantation group, the amount of total collagen and type I collagen was higher than those in unimplanted controls.

## Discussion

Ideal implant biomaterials should induce osteogenesis in vivo by promoting osteogenic differentiation of BMSCs. In this study, BMSCs and MLO-Y4 were spheroid co-cultured in different proportions to promote the osteogenic differentiation of BMSCs and yield a bone-like tissue with internal structure and mineral composition similar to mouse femur. Among the different co-culture ratios, the BMSC:MLO-Y4 3:1 co-culture yielded a ring-shaped bone-like tissue that was the most stable and similar to mouse femur. The ring-shaped bone-like tissue accelerated tooth extraction socket healing by promoting bone and collagen formation in vivo.

In bone tissue engineering, the application of 3D culture in the formation of biomaterials has gained increasing attention. Spheroid culture is a promising method that offers the advantage of maintaining the survival, stemness, and differentiation potential of cells while providing an in vivo-like microenvironment by promoting cell–cell and cell–matrix interactions^16–18^. Our findings demonstrate that spheroid culture is an effective method for developing bone-like tissue (Fig. 1). As an important source of seed cells in bone tissue engineering, BMSCs have the potential to differentiate into osteogenic, chondrogenic, and adipogenic cells^35^. However, they will differentiate into osteoblastic lineage cells only under osteogenic culture conditions, so an appropriate microenvironment is needed when using BMSCs for tissue engineering. In our study, we co-cultured different ratios (3:1, 2:1, 1:1, 1:2, and 1:3) of BMSCs and murine MLO-Y4 osteocyte-like cells, which shares many characteristics with primary osteocytes^36^. We found that the 3:1, 2:1, and 1:1 co-cultures and the MLO-Y4 monoculture formed bone-like tissues (Fig. 1a,b), while the 3:1 co-culture implantation group showed more type I collagen which was a major component of ECM in bone than in unimplanted controls (Fig. 4g). This may be related to the interaction between two or more cell types can promote ECM remodeling, and ECM from multiple cell types better simulates a tissue microenvironment than ECM from a single type^37,38^.

Although BMSCs have been widely applied in bone tissue engineering, the results of a previous study indicated that the formation of bone-like tissue with specific shapes relies on an exogenous scaffold-based culture model^39^. Of interest, the bone-like tissue formed at the 3:1 ratio in the current study was ring-shaped despite the absence of a scaffold. With a prolonging of the culture time, the area of the bone-like tissue in the 3:1 co-culture showed no evident shrinking and still adhered to the bottom of the dish on day 49 (Fig. 1c). In contrast, the areas of the bone-like tissues in the 2:1 and 1:1 co-cultures shrank gradually and separated from the culture dishes on day 49. These results indicated that a ring-shaped bone-like tissue is more stable than the tissues that do not take this shape, which is to be expected: the spheroid culture has the physical limitation that nutrients, oxygen, and waste cannot diffuse through the spheroid interior^17^, and a ring shape can effectively solve this shortcoming. These results preliminarily demonstrate that a 3:1 ratio of BMSCs to MLO-Y4 cells is best for bone-like tissue formation and that a proper ratio of BMSCs and MLO-Y4 cells is essential for the function of the co-culture.

To explore the similarity between bone-like tissue cultured in vitro and bone tissue in vivo, we used SEM and EDX analysis to compare the bone-like tissue and mouse femur, and immunohistochemical analysis to detect osteoblast markers and osteocyte markers in cells of bone-like tissue. SEM showed continuous dense calcium phosphate structures and ECM similar to the cortical bone of mouse femur on the surface of the bone-like tissue (Fig. 2). Spherical calcium phosphate similar to trabecular bone also was found in cross-sections of the bone-like tissue in the 3:1, 2:1, and 1:1 co-cultures (Fig. 2). Using 3D-cultured bone-like tissue in gelatin hydrogels, Takagishi et al. also observed a dense layer structure and spherical calcium phosphate similar to cortical bone and trabecular bone of mice^40^. In addition, flake-like calcium phosphate was present in the bone-like tissues of the 3:1 and 2:1 co-cultures in the current work (Fig. 2b2,c2). In the study using seeding of human osteoblast-like MG-63 cells on 3D scaffolds reinforced with zinc oxide, Feng et al. noted the appearance of spherical and flake-like calcium phosphate on days 7 and 21 of culture, and showed that the flake-like structures were more mature^41^. In line with the SEM surface morphology findings in the current study, the elemental analysis showed that the P and Ca content was highest in the 3:1 co-culture (Table 1). Taken together, these results indicate that the internal structure and mineral composition of bone-like tissue in the 3:1 co-culture were the most similar to the mouse femur.

HE staining showed that the bone-like tissue of the 3:1 co-culture had an osteoid-like matrix encircling osteocyte-like cells, similar to bone, and also had the largest area of any of the cultures (Fig. 3a). Further immunohistochemical analysis demonstrated that the osteocyte-like cells embedded in the osteoid-like matrix had osteocyte features in vivo, expressing the osteocyte marker DMP1 and not expressing the osteoblast markers ALP and Col1 (Fig. 3)^29^. Moreover, osteoblast-like cells at the edge of the osteoid-like matrix expressed ALP and Coll but not DMP1. These results further suggest that the bone-like tissue from the 3:1 co-culture is highly similar to bone in vivo. Osteocyte-like cells in the osteoid-like matrix were GFP positive, indicating that this bone-like tissue originated from osteogenic differentiation of GFP^+^BMSCs, whereas the MLO-Y4 cells played an auxiliary role in the formation of this tissue. This pattern is consistent with findings of a previous study showing that BMSCs are the ideal seed cells for bone tissue engineering^21^ and studies showing that osteocytes can promote the osteogenic differentiation of BMSCs^31,32^.

To assess the effect of bone-like tissue on bone regeneration in vivo, we transplanted the ring-shaped bone-like tissues into tooth extraction sockets of mice. The implantation group developed more bone mass in the extraction sockets than the unimplanted control group, indicating that this bone-like tissue could promote bone regeneration in vivo. Barati et al. found that a ring-shaped scaffold mimicking the hierarchical structure of cortical bone could induce BMSCs osteogenesis and vasculogenesis of endothelial colony-forming cells in the absence of bone morphogenetic proteins^42^. Consistent with the above work, the presence of more osteoid and mineralized bone in the bone-like tissue implantation group in our study demonstrated that ring-shaped bone-like tissue promotes bone formation in vivo (Fig. 4e). To reveal the ECM differences between the two groups, we used picrosirius red staining. Compared with unimplanted controls, the bone-like tissue implantation group had a higher ECM content, including more total collagen and type I collagen (Fig. 4f,g). The increase in ECM may be partially because cell aggregates of spheroid culture would mostly reserve the ECM^43^. As an important organic component of bone tissue, ECM can regulate cell behavior by affecting cell–ECM interaction and directing the tissue regeneration process^27^. We previously found that collagen type I alpha2 (COL1A2), an important component of bone ECM, was elevated in *Irs-1*-null mice, thus promoting osteogenic differentiation of BMSCs^29^. Moreover, up-regulation of COL1A2 protein expression promotes osteoblast differentiation of primary pre-osteoblasts^44^. Thus, increased ECM in the bone-like tissue implantation group would be expected to promote bone formation. In the process of bone formation, type III collagen, representing new bone, would form initially and then be replaced by type I collagen, representing mature bone^45^. The higher content of type I collagen in the bone-like tissue implantation group in the current study indicates that this tissue could promote bone maturation. Thus, the in vivo results showed that the ring-shaped bone-like tissue could accelerate alveolar bone regeneration by promoting bone formation and maturation.

## Conclusions

In this study, bone-like tissue with an internal structure and mineral composition similar to mouse femur was formed via spheroid co-culturing of BMSCs and MLO-Y4 cells. A 3:1 co-culture ratio of BMSC:MLO-Y4 resulted in the formation of a ring-shaped bone-like tissue that was most similar to the in vivo bone. The implantation of this tissue into tooth extraction sockets of mice demonstrated that it could promote bone formation and maturation, thus accelerating alveolar bone regeneration. Our findings point to the potential for in vitro fabrication of bone-like tissue to mimic in vivo bone, opening the way to the development of new bioactive materials and treatment strategies for bone tissue engineering.

## Materials and methods

### Cell culture and formation of bone-like tissue

The mouse osteocyte-like cell line MLO-Y4 was purchased from American Type Culture Collection (Manassas, VA, USA). Mouse GFP^+^BMSCs were a gift from Chang-Jun Li (Central South University, Changsha, China)^46^. To form bone-like tissues, we used the spheroid culture method as previously published^47^. Briefly, the cells (10^6^ cells/well) were suspended and mixed in complete culture medium constituted by alpha minimum essential medium (Gibco) supplemented with 10% fetal bovine serum (Gibco) and 1% penicillin–streptomycin (Gibco), and then pelleted by centrifugation at 180×*g* for 5 min and cultured in 6-well plates at 5% CO_2_ at 37 °C for 49 days. This study included seven groups: five co-culture groups (GFP^+^BMSC: MLO-Y4 in ratios of 3:1, 2:1, 1:1, 1:2, and 1:3; n = 3) and two monoculture groups (GFP^+^BMSCs alone and MLO-Y4 cells alone; n = 3).

### SEM and EDX analysis

SEM (FEI Company, Hillsboro, OR, USA) analysis was performed to evaluate the morphology of bone-like tissues and mouse femoral bone (n = 3). Samples were fixed with 1% formaldehyde solution for 24 h. The substrates were then washed in distilled water and dehydrated through graded ethanols. Specimens were sputter-coated with gold and then observed by SEM and analyzed using EDX software.

### Tooth-extraction mouse model

Eight-week-old male C57BL/6 mice were purchased (Hunan SJA Laboratory Animal Co., Ltd., Changsha, Hunan, China) and kept under specific-pathogen-free conditions (n = 3). After animals were anesthetized with 1% pentobarbital sodium (50 mg/kg), the bilateral maxillary first molars were extracted. In each animal, the extraction sockets of the left first molar were immediately implanted with one ring-shaped bone-like tissue formed in the 3:1 co-culture group with the right side as the untreated control. After 2 weeks, the mice were sacrificed and the maxillae collected. The protocol for the mouse experiments was approved by the Animal Ethics Committee of the Second Xiangya Hospital of Central South University. All animal experiments were conducted in accordance with the relevant national guidelines and the ARRIVE guidelines.

### Micro-CT

The maxillae were fixed with 4% paraformaldehyde for 48 h and stored in 70% ethanol at 4 °C. For analyses, the maxillae were scanned using a micro-CT Scanner (eXplore Locus SP, GE Healthcare, USA) at a resolution of 8 µm, a voltage of 80 kV, and a current of 80 μA. The region of interest was selected in the defect area after 3-dimensional image reconstruction. The quantitative analysis was performed using V.G studio (version 3.0; Volume Graphics GmbH, Heidelberg, Germany). The evaluated morphometric parameters of trabecular bone in the extraction sockets were the fraction of bone BV/TV, Tb.Th, Tb.N, and Tb.Sp.

### Histomorphological and immunohistochemical analysis

Bone-like tissues and maxillae were fixed in 4% paraformaldehyde for 48 h. After being deparaffinized with xylene and rehydrated with a graded series of alcohol solutions, the 4-μm-thick sections were stained with HE according to the method of Wu et al.^48^. To determine the location of GFP^+^BMSCs in bone-like tissues, samples dyed with 4′,6-diamidino-2-phenylindole were observed under a fluorescence microscope (Leica, Wetzlar, Germany). ALP staining was performed according to our previously published method^29^. For immunohistochemical analyses, sections were deparaffinized and heat treated for antigen retrieval. The activity of endogenous peroxidase was blocked by 0.3% H_2_O_2_ in phosphate-buffered saline. After a treatment with 0.1% trypsin for 30 min, the sections of bone-like tissues were incubated at 4 °C overnight with a Col1 antibody (NB600-408, 1:100, Novus Biologicals, Littleton, CO, USA) and a DMP1 antibody (sc-54181, 1:100; Santa Cruz Biotechnology, Santa Cruz, CA, USA) followed by incubation with secondary antibodies. Antibodies were detected by staining with a horseradish peroxidase-conjugated anti-mouse/rabbit IgG and diaminobenzidine (GTVision III Detection System/Mo&Rb Kit; Gene Tech, Shanghai, China) and the UltraSensitive S-P Kit (Maxin Biotechnology Ltd., Fuzhou, China), respectively. Specimens were then re-stained with hematoxylin. Goldner’s trichrome staining^49^ and picrosirius red staining^50^ were performed according to a standard protocol. After being fixed in 4% formaldehyde for 48 h, the undecalcified sections of the maxillae were stained with picrosirius red and Goldner’s trichrome according to the manufacturer’s instructions (Servicebio, Wuhan, China). Then, Goldner’s trichrome staining was observed by ordinary white-light microscopy, and picrosirius red staining was observed by both ordinary white-light microscopy and polarized light microscopy.

### Statistical analyses

SPSS 19.0 and GraphPad Prism 9.0 software were used for data analysis. A Kolmogorov–Smirnov test was performed to evaluate the normal distribution of the data. A two-tailed paired Student’s t-test was used to analyze differences between two groups. *P* < 0.05 indicated statistical significance.

### Ethics approval

The protocol for the mouse experiments was approved by the Animal Ethics Committee of the Second Xiangya Hospital of Central South University. All animal experiments were conducted in accordance with the relevant national guidelines and the ARRIVE guidelines.

## Data Availability

The data sets used and/or analyzed during the current study are available from the corresponding author on reasonable request.

## Abbreviations

GFP: Green fluorescent protein
BMSCs: Bone marrow stromal cells
SEM: Scanning electron microscopy
3D: Three-dimensional
ECM: Extracellular matrix
hPDL: Human periodontal ligament
MSCs: Mesenchymal stem cells
ALP: Alkaline phosphatase
BV/TV: Fraction of bone volume/total volume
Tb.Th: Trabecular thickness
Tb.N: Trabecular number
Tb.Sp: Trabecular separation
HE: Hematoxylin–eosin
Col1: Type I collagen
DMP1: Dentin matrix protein 1
C: Carbon
O: Oxygen
P: Phosphorus
Ca: Calcium
